# Applications of social constructivist learning theories in knowledge translation for healthcare professionals: a scoping review

**DOI:** 10.1186/1748-5908-9-54

**Published:** 2014-05-06

**Authors:** Aliki Thomas, Anita Menon, Jill Boruff, Ana Maria Rodriguez, Sara Ahmed

**Affiliations:** 1 School of Physical and Occupational Therapy, McGill University, Montreal, Quebec, Canada; 2 Centre for Interdisciplinary Rehabilitation Research of Greater Montreal, Montreal, Quebec, Canada; 3 Centre for Medical Education, Faculty of Medicine, McGill University, Montreal, Quebec, Canada; 4 Life Sciences Library, Faculty of Medicine, McGill University, Montréal, Quebec, Canada

## Abstract

**Background:**

Use of theory is essential for advancing the science of knowledge translation (KT) and for increasing the likelihood that KT interventions will be successful in reducing existing research-practice gaps in health care. As a sociological theory of knowledge, social constructivist theory may be useful for informing the design and evaluation of KT interventions. As such, this scoping review explored the extent to which social constructivist theory has been applied in the KT literature for healthcare professionals.

**Methods:**

Searches were conducted in six databases: Ovid MEDLINE (1948 – May 16, 2011), Ovid EMBASE, CINAHL, ERIC, PsycInfo, and AMED. Inclusion criteria were: publications from all health professions, research methodologies, as well as conceptual and theoretical papers related to KT. To be included in the review, key words such as constructivism, social constructivism, or social constructivist theories had to be included within the title or abstract. Papers that discussed the use of social constructivist theories in the context of undergraduate learning in academic settings were excluded from the review. An analytical framework of quantitative (numerical) and thematic analysis was used to examine and combine study findings.

**Results:**

Of the 514 articles screened, 35 papers published between 1992 and 2011 were deemed eligible and included in the review. This review indicated that use of social constructivist theory in the KT literature was limited and haphazard. The lack of justification for the use of theory continues to represent a shortcoming of the papers reviewed. Potential applications and relevance of social constructivist theory in KT in general and in the specific studies were not made explicit in most papers. For the acquisition, expression and application of knowledge in practice, there was emphasis on how the social constructivist theory supports clinicians in expressing this knowledge in their professional interactions.

**Conclusions:**

This scoping review was the first to examine use of social constructivism in KT studies. While the links between social constructivism and KT have not been fully explored, the Knowledge to Action framework has strong constructivist underpinnings that can be used in moving forward within the broader KT enterprise.

## Introduction

Third party payers, insurers, professional regulatory boards, and patients increasingly expect healthcare professionals to integrate new knowledge and scientific evidence into daily practice [1,2], with the ultimate goal of increasing their use of evidence-based practice (EBP) [3]. EBP has been shown to have a direct impact on improving patient outcomes [4].

Despite clear advantages for adhering to EBP principles, not all health professionals readily integrate scientific evidence into clinical decision making [5]. In the Netherlands and the United States, it is estimated that 30% to 45% of patients are not receiving care according to scientific evidence, and that 20% to 25% of the care provided is often unnecessary or potentially harmful [6,7]. In Canada, research studies in stroke rehabilitation have indicated that clinicians fail to routinely apply best practices [8-10]. For example, in a multi-center study of stroke rehabilitation therapists, Menon, Korner-Bitensky and Ogourtsova [11] found that only 13% of patients with unilateral spatial neglect (USN) post-stroke were assessed or screened with a standardized USN-specific tool during their acute care admission.

Recognition of the gap between what is known to improve patient outcomes and what is used in daily practice has led to a growing interest in knowledge translation (KT), defined as the exchange, synthesis and ethically sound application of knowledge to improve health and provide more effective health services [12]. Developing effective KT interventions that maximize clinicians’ knowledge about best practices is an important step towards closing this knowledge-to-practice gap.

Some have argued that the use of theory is essential for advancing the science of KT and for increasing the likelihood of successful KT interventions for reducing these practice gaps [13-15]. Indeed, this is similar to the Medical Research Council’s framework for the design of complex interventions, which stresses the importance of theory as a central part of designing, and testing interventions [83]. Greater use of theory can lead to a greater understanding of barriers and enablers of behavior change, inform the design of KT interventions, and allow for exploration of causal pathways and moderators for successful application of EBP [15]. Eccles *et al.*[14] highlighted how theories can be used to help design KT interventions and understand their impact on individuals and team behaviors. They emphasized that two objectives should be considered for the application of theories. The first objective is ‘to develop an understanding of the theory-based factors that underlie clinical practice and to identify theoretical constructs that are important for current patterns of care- these should be the targets of a KT intervention’ (p.3). This implies that theories could shed light on the multiple variables (both individual and organizational) that influence clinical behaviors, so that appropriate and targeted interventions can be designed to influence the likelihood that a given stakeholder will adopt a desired behavior. The second objective is ‘to develop/test KT interventions that target specific theoretical constructs and to design these interventions for enhancing the processes that support change in them’ [14] (p.3). While Eccles *et al.* (2005) and others [16,17] recommend a more systematic use of theory to increase the chances of successful implementation, theories have been rarely used to inform the design and evaluation of KT interventions [5,18]. This observation was recently corroborated by Colquhoun *et al.*[19] and Davies, Walker, and Grimshaw [20] who also reported a limited use of KT theories, along with broader paradigms such as social cognitive theory, learning theories, and organizational theories. Colquhoun *et al.*[19] indicated that theories in KT studies tend to be mostly used in the fields of medicine and nursing, mainly to predict the success of KT interventions. A review by Davies *et al.*[20] found that only 6% of included studies used theory to inform the design and/or the implementation of KT interventions. Most were theories of behavior or behavior change, including: ‘diffusion of innovation’, ‘the theory of reasoned action’, ‘health beliefs model’, and ‘organizational development’. The review identified a number of studies reporting on KT interventions underpinned by two broad categories of theories: cognitive theories (*e.g.*, social cognitive theory) and theories of learning (*e.g.*, social learning theory). None of the studies reviewed were grounded in social constructivist theory [20].

### Potential application of social constructivist theories in KT

Several authors conceptualize KT as a process that occurs through social and environmental interactions, and emphasize that knowledge exchange between researchers and healthcare professionals must happen in a mutually created social context [21,31-33]. Indeed, knowledge use within KT can be regarded as an active learning process, because knowledge is not an inert object to be ‘sent’ and ‘received’, but a fluid set of understandings shaped by those who produce it and those who use it. Clinicians act upon new knowledge by transforming the information based on pre-existing experiences and understandings, by relating it to existing knowledge, imposing meaning to it and, in many cases, monitoring their understanding throughout the process. Hence, the meaning of research is constructed by the user and casts the clinician as an active problem solver and a constructor of his or her own knowledge, rather than a passive receptacle of information [22]. This has led us to propose that social constructivist theory may be useful for understanding why and how individuals integrate and apply new knowledge in evidence-based clinical decision making and how practice behaviors may change as a result of KT interventions grounded in the core tenets of this theory. We wish to emphasize that in this paper, we are focusing on constructivism, not constructionism. Though the two terms tend to be used interchangeably and often unapologetically [84], p.30, they are not synonyms. Social constructionism emphasizes purposeful creation of knowledge. The focus is on revealing the ways in which individuals and groups participate in the creation of their perceived social reality. It involves looking at the ways social phenomena are created, institutionalized and made into tradition by humans. Socially constructed reality is seen as an ongoing, dynamic process, and reality is reproduced by individuals acting on their interpretation and their knowledge. According to Burr (2003) there is no one feature which could be said to identify a social constructionist position, but there are assumptions among individuals who identify as such namely, ‘a critical stance towards taken-for granted knowledge, historical and cultural specificity, knowledge is sustained by social processes and knowledge and social action go together’ [85]. The social context is at the center of ‘meaning making’ in social constructionism and the attention is on the ‘knowing’ that is created through shared production. Constructionism also ‘emphasizes the hold our culture has on us: it shapes the way in which we see things and gives us a quite definitive view of the world’ [86] (p.58). In contrast, within a social constructivist paradigm, the individual is at the center of the meaning making experience. The focus of constructivism is on the individual’s learning that takes place because of their interactions within a particular social context. According to Crotty (1998), ‘it would appear useful, then, to reserve the term constructivism for epistemological considerations focusing exclusively on ‘the meaning making activity of the individual mind’ and to use constructionism where the focus include the collective generation [and transmission] of meaning’ [86] (p.58). We privileged social constructivism as the focus of this review, for its emphasis on the individual and how/she he creates knowledge in socially medicated contexts.

Social constructivism is a sociological theory of knowledge that focuses on how individuals come to construct and apply knowledge in socially mediated contexts [21,22]. The fundamental premise of this theory is that knowledge is a human construction and that the learner is an active participant in the learning process [23]. Constructivism is based on three assumptions about learning [24-28]. First, learning is a result of the individual’s interaction with the environment. Knowledge is constructed as the learner makes sense of their experiences in the world. The content of learning is not independent of how the learning is acquired; what a learner comes to understand is a function of the context of learning, the goals of the learner, and the activity the learner is involved in. Second, cognitive dissonance, or the uncomfortable tension that comes from holding two conflicting thoughts at the same time, is the stimulus for learning. It serves as a driving force that compels the mind to acquire new thoughts or to modify existing beliefs in order to reduce the amount of dissonance (conflict). Cognitive dissonance ultimately determines the organization and nature of what is learned [29]. Third, the social environment plays a critical role in the development of knowledge. Other individuals in the environment may attempt to test the learner’s understanding and provide alternative views against which the learner questions the viability of his knowledge. Constructivism supports the acquisition of cognitive processing strategies, self-regulation, and problem solving through socially constructed learning opportunities [25,26,28,30], all of which are critical skills for evidence-based knowledge uptake and implementation in clinical practice [31].

The Knowledge to Action (KTA) framework [32] adopted by the Canadian Institutes for Health Research, is a widely used framework that focuses on knowledge creation and exchange. The KTA framework contains two principal components, a knowledge creation funnel and an action cycle. The knowledge creation funnel consists of three phases: knowledge inquiry, knowledge synthesis, and knowledge tools and products. The action cycle consists of seven stages involved in moving knowledge into practice: identifying a problem in practice or a gap in knowledge and identifying, reviewing, and selecting the knowledge to be implemented to address the gap; adapting or customizing the knowledge to the local context; evaluating the determinants of the knowledge use (barriers and facilitators); selecting, tailoring and implementing interventions to address the knowledge or practice gap; monitoring the knowledge use in practice; evaluating the outcomes or impact of using the new knowledge; and determining strategies for ensuring that the new knowledge is sustained [32]. The KTA framework is grounded in the social constructivist paradigm which privileges social interaction and adaptation of research evidence by taking the local context and culture into account [34]. To our knowledge, this is the only KT framework developed with social constructivist underpinnings. Despite the growing recognition that the KTA framework can facilitate knowledge use and exchange in practice, its association with social constructivist theory has yet to be explicitly explored.

Social constructivist approaches to the science of KT have the potential to support researchers interested in examining how learning in the clinical context occurs and how new knowledge is created, disseminated, exchanged and used to inform practice. While social constructivist theory may be useful for informing the design and evaluation of KT interventions, we have yet to understand the extent to which social constructivist theory has been applied in the KT literature for healthcare professionals. Thus, this paper presents the results of a scoping review on the application of social constructivist theory in KT for healthcare professionals.

## Methods

There are four reasons for undertaking scoping reviews: to examine the extent, range and nature of research activity, to determine the value of undertaking a systematic review, to summarize and disseminate research findings, and to identify research gaps in the existing literature [35]. The objectives of the scoping review reported in this paper were to summarize and disseminate findings from a broad body of literature and identify research gaps in the existing literature. Using the Arksey and O’Malley framework [35], we outline the specific methods for our scoping review below:

### Step one: research question

The research question that guided the review was ‘What are the applications of social constructivism and/or social constructivist theories in KT to promote EBP among healthcare professionals’? We used the PICOS format as a structure for our research question and to design our search strategy. The population is ‘healthcare professionals’; intervention is ‘application of social constructivism in KT’; the outcome is ‘promote EBP’; and the study design refers to all the study designs eligible for inclusion in the review. Eligible study designs included: all qualitative methodologies and quantitative designs (observations studies, randomized controlled trials, cohort studies, cross sectional studies, longitudinal studies and case studies).

### Step two: identifying relevant studies and study selection

All members of the research team were involved in decisions about inclusion and exclusion criteria. The team worked with a rehabilitation sciences librarian (JB) who suggested that, given our research question, we take a broad approach to the concept of KT when selecting search terms. The terms captured both the theory and application of KT as discussed by McKibbon [36], and took into account the terms used by a previous systematic review on KT in rehabilitation [37]. A first pilot search was constructed to include articles where any variation of the word ‘constructivism’ or ‘constructivist’ appeared in the title or abstract of articles discussing health professionals. A research assistant under the supervision of one member of the research team (AT) was responsible for reading the abstracts of all the articles identified in this first search and applying the original inclusion/exclusion criteria in an abstract screening tool. Two members of the research team (AT and AM) piloted the inclusion/exclusion criteria with a subset of abstracts retrieved from MEDLINE. The same two members of the research team (AT and AM) reviewed the search terms, the redesigned strategy and approved the abstract screening tool. This process resulted in modifications to the inclusion/exclusion criteria and the search was redesigned to include: publications from all health professions; all research methodologies (quantitative and qualitative); conceptual and theoretical papers related to KT; and, papers written in English. Excluded from the review were papers that had no evidence of the concept of knowledge translation in the abstract; were unrelated to any health profession or health field; discussed new curricula designed to promote higher level learning in health sciences students; and described new pedagogical methods (*i.e.*, virtual, simulating techniques, etc.) for teaching in schools.

The pilot search developed for MEDLINE was conducted again with the new inclusion criteria, and then adapted for other databases. The Ovid MEDLINE search strategy is presented as ‘Additional file 1’. Searches were conducted in six databases: Ovid MEDLINE (1948 – May 16, 2011), Ovid EMBASE (1980 – May 16, 2011), CINAHL (searched entire database to May 16, 2011), ERIC (1966 – May 16, 2011), PsycInfo (1967 – May 16, 2011), and AMED (1985 – May 16, 2011). The librarian used GoPubMed to analyze the subject headings of the full-text articles that were assessed and considered for eligibility (see PRISMA flow chart) and then again of the final articles to determine whether any important terms had been missed. The iterative nature of scoping reviews allowed the research team to consider the addition of articles that best reflected new ideas gained from the review process. The GoPubMed analysis added seven other medical subject headings to the search (line 21 in Additional file 1). An expanded search including these new subject headings was conducted six months later.

### Step three: charting the data

The authors developed a data charting form that included the following categories: author, year of publication, purpose of the study/research question, practice setting, nature of theory use, links with the KTA framework, methodology, population characteristics, outcome evaluation (evaluation setting, evaluation responses, effectiveness of implementation, variables of evaluation, outcomes), implications for practice, and directions for future research. The data charting form was piloted on the first 10 articles and reviewed by the research team to ensure that it was comprehensive. A research assistant extracted the data for the remaining articles. The two senior authors (AT and SA) reviewed and discussed the completed extraction tables. Categories such as ‘population characteristics’, ‘outcome evaluation’, ‘effectiveness of implementation’, and ‘evaluation variables’ were not appropriate for several conceptual papers and were adapted to be more inclusive. This was an iterative process that ensured that the tables included all the salient information for generating the themes as per step four described below.

### Step four: collating, summarizing and reporting the results

An analytical framework of quantitative (numerical) and thematic analysis was used to examine and combine study findings [35]. The numerical analysis highlighted: the nature and distribution of the studies; the nature of the social constructivist assumptions used in each study; and the KTA stage/component targeted.

The nature of the application or use of social constructivism across all papers served as the major unit of analysis. We also aimed to identify which of the three social constructivist assumptions were used in the selected studies. Two members of the research team (AT and SA) independently reviewed the data charting tables and identified a number of preliminary emerging themes. All other members of the research team were consulted to discuss the themes and ensure agreement. This process resulted in the generation of five themes. With the assistance of a doctoral student (AMR), we revisited all the charting tables to confirm that these corresponded with the themes that were generated. A summary of the major findings organized under each theme was produced following several iterations and meetings with the research team.

## Results

### Nature and distribution of the studies

**Figure 1:**
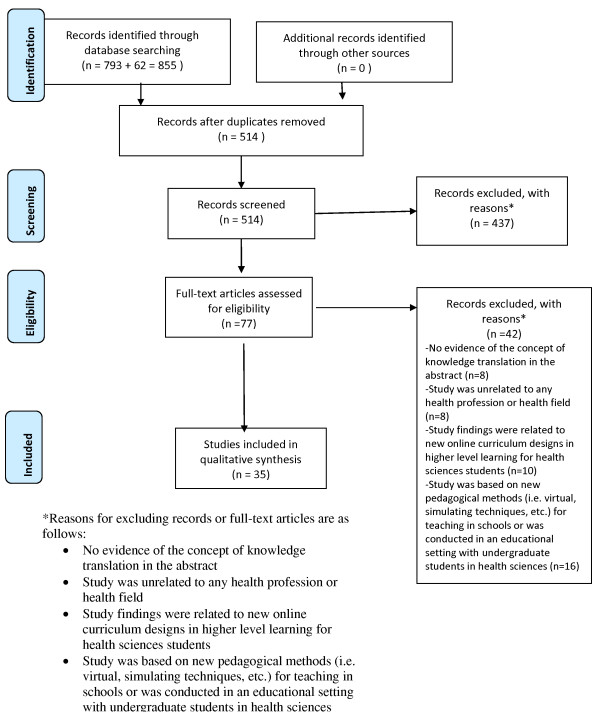
**PRISMA Flow Diagram.** *Reasons for excluding records or full-text articles are as follows: no evidence of the concept of knowledge translation in the abstract; study was unrelated to any health profession or health field; study findings were related to new online curriculum designs in higher level learning for health sciences students; study was based on new pedagogical methods (*i.e.*, virtual, simulating techniques, etc.) for teaching in schools or was conducted in an educational setting with undergraduate students in health sciences.

A total of 855 results were retrieved from all sources. Duplicates were removed (n = 341), yielding 514 records for eligibility screening. We screened the 514 abstracts and excluded 437 papers on the basis of our four exclusion criteria. Seventy-seven articles were read in full and assessed for eligibility. Fourty-two additional papers were excluded for the following reasons: no evidence of the concept of knowledge translation in the abstract (n = 8); study was unrelated to any health profession or health field (n = 8); study findings were related to new online curriculum designs in higher level learning for health sciences students (n = 10); study was based on new pedagogical methods (*i.e.*, virtual, simulating techniques, etc.) for teaching in schools or was conducted in an educational setting with undergraduate students in health sciences (n = 16). The number of eligible article at this stage was 35. The expanded search resulted in an additional 55 additional articles plus seven MEDLINE articles for screening for a total of 62 additional articles for screening. In the end however, none of these new articles from the expanded search were eligible for the final review. The numbers of articles at each stage selection process are shown in the PRISMA flow chart (Figure 1).

Thirty-five papers published between 1992 and 2011 met the inclusion criteria. Table 1 shows the charting categories and associated content for the 35 studies. Tables 2, 3, 4 represent the study designs, practice setting, and professional groups respectively. Twenty-seven studies used a qualitative study design. These ranged from various types of literature reviews, conceptual and reflective papers to studies using interviews and questionnaires, focus groups and observations. Six papers described the results of KT interventions that for the most part, consisted of a workshop or a didactic course [38-43]. Two studies used a mixed method design [44,45] (Table 2). The most common practice settings identified were primary healthcare (n = 10) [42,44,53,59-62,64,65,82], followed by post-graduate educational settings (n = 7) [39-41,43,45,65,81], and mental health clinical environments (n = 5) [50,52,58,78,80] (Table 3). Nursing was the professional group most frequently targeted in the papers (20 of 35 included studies), alone [42,43,47,53,55,60-62,77], or along with physicians [64,82], patients [44], or interdisciplinary teams [38,39,48,51,57,65,76,79]. Psychologists/psychiatrists was another identified group (n = 5) [50,52,58,78,80]. Four papers [40,41,45,80] presented results of studies conducted with postgraduate (*e.g.*, residents and other trainees not considered undergraduate learners) health care professionals (Table 4).

**Table 1:** Descriptive information for each study included in the scoping review

**First author (Year)**	**Main theme**	**Area of practice**	**Target population**	**Study design**	**Intervention/approach**	**Main findings**	**Theory described**	**Theory integrated**
**Abad-Corpa (2010)**[44]	Design of a KT activity/intervention (to improve EBP in nurses and outcomes in patient)	Primary health care setting	- Nurses	Mixed (qualitative approach, quantitative analysis)	Focus groups (reviewed articles, videos, field diaries, statistics)	-Psycho-social adjustment	Yes	yes
			- Patients (with compromised immune system)					
						-Satisfaction with nursing		
						-Family burden		
**Adler (2002)**[46]	Meaning of ‘evidence’, tension between research and practice	N/A	Focus on Physicians involved in research	Qualitative	Review of the literature and history of science	Reflection on the type of evidences to use in health research	Yes	N/A
**Appleton (2002)**[47]	Meaning of ‘evidence’, tension between research and practice	N/A	Focus on Health services researchers	Qualitative	Review of the types of philosophical approaches and reflection on the implication for practice	Philosophical underpinnings of constructivism and relevance to researchers in health services	Yes	N/A but links with health research emphasized
**Carr (2005)**[54]	Acquisition, expression and application of knowledge for professional practice	N/A	Focus on nurses	Qualitative	Reflective/guidance elaboration	The interpretive paradigm provides one means of voicing nursing knowledge.	Briefly	N/A
**Caley (2010)**[38]	Design of a KT activity/intervention	Health and human services organization	Health and Human service professionals	Intervention	Workshop on alcohol dependence screening, survey	Number of interventions implemented	Yes	Yes
**Cronin (2007)**[45]	Design of a KT activity/intervention	Education (post-graduate training of health care professionals)	Post-graduate health promotion education	Intervention	Workshop on experiential learning, reflective practice, satisfaction survey	Student satisfaction	Yes in part	Yes
**Daley (2001)**[39]	Acquisition, expression and application of knowledge for professional practice	Education (post-graduate training of health care professionals)	Social workers, lawyers, nurses, educators	Intervention	Post-graduate course followed by survey	Identification of key components that made knowledge useful	Yes	Yes
**Fagan(1998)**[62]	To better understand clients, and their experiences/realities	Primary health care setting	Emergency nurses	Qualitative	Questionnaire	Perception of nurses regarding their roles in identifying child abuse	No	No
**Fairweather (2000)**[61]	Learning in promoting professional expertise	Primary health care setting	Specialist nurses	Qualitative	Focus groups	Roles and attributes of specialist vs generalist nursing	Yes	Yes in part
**Felton (2003)**[76]	Meaning of ‘evidence’, tension between research and practice	Community services	Mental health, social services, community services, hospital administrators involved in shelters and housing accessibility	Qualitative	Interview	Consensus on system-level concerns regarding involvement of outside agency in ‘Housing first’ projects	Yes	Yes
**Field (2004)**[55]	Acquisition, expression and application of knowledge for professional practice	N/A	Focus on Nurses	Qualitative	Literature review	Importance of context in learning and difficulty of transferring knowledge to different context	Yes	N/A
**Fonville (2002)**[53]	Acquisition, expression and application of knowledge for professional practice	Primary health care setting	Nurse executives	Qualitative	Interview	Nursing are more loyal to their professional than their organizational entity, unaware of ethics principles, need for reflective learning.	Yes in part	Yes
**Greenhalgh (2006)**[65]	Design of a KT activity/intervention	Education (post-graduate training of health care professionals)	Senior professionals: senior partners in general practice, postgraduate tutors, service managers	Online course	Student Course Evaluation	Web-based learning offers potential for students to engage in rich and effective construction of knowledge.	Briefly	No
**Greenslade (2010)**[63]	To better understand clients, and their experiences/realities	Primary health care setting (same-day surgery)	Breast cancer surgery patients	Qualitative	Interview	Follow-up visit for assessment, education, and psychosocial support recommended.	No	No
**Higgs (1995)**[56]	Acquisition, expression and application of knowledge for professional practice	N/A	Focus on Physical Therapists	Qualitative	Literature review	Knowledge is an active and dynamic phenomenon undergoing constant changes and testing	Yes	N/A
**Holtslander (2008)**[77]	Acquisition, expression and application of knowledge for professional practice	N/A	Focus on palliative nurses	Qualitative	Reflective paper	Exposition of the ways to acquire knowledge and the nursing model in palliative setting	Briefly	N/A
**Hoshmand (1992)**[52]	Meaning of ‘evidence’, tension between research and practice	N/A	Focus on psychological Sciences	Qualitative	Literature review	Emphasis on broadened choices of research methods, the development of reflective skills, and better linkage between teaching in the domains of research and practice are urged.	yes	N/A
**Hunter (2008)**[40]	Design of a KT activity/intervention	Education (post-graduate training of health care professionals)	Nurses	Intervention	Course and Student Course Evaluation and students’ cultural competence levels evaluations	Students’ comments were all positive or politely constructive, their competency increased.	Yes	Yes
**Kinsella (2010)**[57]	Acquisition, expression and application of knowledge for professional practice	N/A	Focus on practice in nursing, health and social care professions	Qualitative	Reflective paper	Discerning philosophical underpinnings of reflective practice to advance increasingly coherent interpretations	Yes	N/A
**Labonte (1996)**[48]	Meaning of ‘evidence’, tension between research and practice	N/A	Focus on health promotion	Qualitative	Literature review	A ‘constructivist’ research paradigm has the potential to resolve some of the tensions between research and practice in health promotion	Yes	N/A
**Lipman (2005)**[59]	Acquisition, expression and application of knowledge for professional practice	Primary health care setting	Physicians researchers in anticoagulation in patients with atrial fibrillation	Qualitative	Interviews	Implementing research evidence is more complex than in suggested in current models of evidence-based medicine	Yes	No
**Lyddon (2006)**[78]	Acquisition, expression and application of knowledge for professional practice	Focuses on counselling (mental health services)	Focus on Psychology	Qualitative	Literature review / reflection	Emerging research strategy in self confrontation method, proven to be a useful procedure for practitioners in counseling settings	Yes	Yes
**McGuckin (2006)**[58]	Acquisition, expression and application of knowledge for professional practice	Not expressed, but most probably mental health services since focus is on psychiatry	Focus on Psychiatry	Qualitative	Literature review / reflection	An eclectic approach that combines elements of the directed approach and the constructivist approach seems warranted	Yes	N/A
**McWilliam (2009)**[79]	Meaning of ‘evidence’, tension between research and practice	Home care programs	Service providers, case managers, administrators, researchers	Qualitative	Action groups to implement KT through social interaction	Sharing accountability for implementation is challenging for achievement-oriented researchers and quality health care practitioners	Yes	Yes
**Miller (2002)**[50]	Meaning of ‘evidence’, tension between research and practice	Not expressed, but most probably mental health services since focus is on psychiatry	Focus on trauma- psychiatry researchers	Qualitative	Literature review / reflection	social constructivism can serve as a bridge between researchers and practitioners by refocusing research efforts to the needs of war-affected communities	Yes	N/A
**Neimeyer (1998)**[80]	Acquisition, expression and application of knowledge for professional practice	Mental health services since focus is on psychiatry	Focus on Psychology-counselling services	Qualitative	Reflection on the literature	Discusses the theories of SC that may support the importation of this theory into the counselling context	Yes	N/A
**Plack (2005)**[49]	Meaning of ‘evidence’, tension between research and practice	N/A	Focus on Physical Therapy	Qualitative	Literature review	PT research should shift its focus from mainly positivism to include constructivism and critical theory for practitioners to better use the evidence	Yes	N/A
**Rogal (2008)**[41]	Design of a KT activity/intervention	Education (post-graduate training of nurses)	Graduate nurses in a Problem-based learning session	Intervention	Course and Satisfaction about education program	Step-by-step guide of constructing a problem based learning package for large, single session groups	Yes	Yes
**Rogers (2011)**[64]	Design of a KT activity/intervention	Primary health care setting	Surgeons and Nurses in OR teams	Qualitative	Focus groups on team conflict	Source of conflict are mainly task-related and concern equipment needs and scheduling. Misattribution and harsh language cause conflict transformation	Very little	Yes
**Rolloff (2006)**[81]	Acquisition, expression and application of knowledge for professional practice	Education (professional training of nurses)	Focus on Nurses	Qualitative	Literature review	A constructivist approach to the baccalaureate nursing curriculum for evidence based practice	Yes	Sometimes referred to
**Smith (2007)**[42]	Design of a KT activity/intervention	Primary health care setting	Nurses	Intervention	Compare 2 instructional design strategies in pain management	Constructivist design took more time, no difference between constructivist and traditional design, learner satisfaction with online experience	Yes	Yes
**Schluter (2011)**[60]	Acquisition, expression and application of knowledge for professional practice	Primary health care setting	Medical and surgical nurses	Qualitative	Interviews	Limits of scope of practice between different nursing practices	Yes	Yes
**Tilleczek (2005)**[43]	Design of a KT activity/intervention	Education (post-graduate training of health care professionals)	Nurses	Intervention	Online course and survey	Increased knowledge and skills, confidence in daily practice. Learners appreciated flexibility of online learning	Yes	No
**Varpio (2006)**[82]	Acquisition, expression and application of knowledge for professional practice	Primary health care setting	Physicians and nurses, both novice and experts using electronic patient records	Qualitative	Non participant observation and interviews	Electronic patient records were printed and the information modified, as it did not facilitate professional work activities.	No	No
**Wilson (2000)**[51]	Meaning of ‘evidence’, tension between research and practice	N/A	Focus on biomedicine	Qualitative	Literature review / reflection	Biomedicine model, debate of effectiveness of objectivism approach in health care vs. subjectivist model, which includes the new emerging theory of SC	Yes	Yes

**Table 2:** Study designs of included studies

**Study design and N of studies**	**Method**	**Number of studies**
**Intervention (mixed experimental)**	Workshop or course followed by survey	5
		[38-41,43]
	Comparison of different learning programs	1
		[42]
**Mixed (qualitative and quantitative)**	Focus Group and quantitative analysis	1
		[44]
	Workshop and reflective practice/discussion	1
		[45]
**Qualitative**	Action Group	1
		[79]
	Focus group	1
		[64]
	Interview/Questionnaire	8
		[53,59-63,65,76]
	Observation/Interview	1
		[82]
**Other**	Editorial Opinion Or Reflective paper	5
		[47,54,57,77,80]
	Literature review	11
		[46,48-52,55,56,58,78,82]
		**TOTAL 35**

**Table 3:** Practice settings of included studies

**Practice settings**	**Number of studies**
Primary health care setting	10
	[42,44,53,59-62,64,65,82]
Health, health promotion, and health services organizations	2
	[38,48]
Post-graduate education	7
	[39-41,43,45,65,81]
Mental Health services	5
	[50,52,58,78,80]
Mental health services, social, and community services	1
	[76]
Home care programs	1
	[79]
Not applicable	9
	[46, 47, 4449, 51 [55-57,77]

**Table 4:** Participants/professional groups of included studies

**Participants/professional groups**	**Number of studies**
Nurses	9
	[42,43,47,53,55,60-62,77]
Nurses and patients	1
	[44]
Nurses and physicians	2
	[64,82]
Interdisciplinary team	8
	[38,39,48,51,57,65,76,79]
Physicians	2
	[46,59]
Health services researchers	1
	[54]
Physical therapists	2
	[49,56]
Psychology/psychiatry health care professionals and researchers	5
	[50,52,58,78,80]
Patients	1
	[63]
Post-graduate (health care professional training)	4
	[40,41,45,80]

### Social constructivist assumptions

Table 5 illustrates that 15 papers discussed research grounded in the social constructivist assumption ‘learning is a result of the individual’s interaction with the environment’. Eight studies corresponded to the assumption that ‘the social environment plays a critical role in the development of knowledge’ and four studies were about ‘cognitive dissonance as the stimulus for learning’. Eight studies explored all three assumptions.

**Table 5:** Aspects of theory used in studies

**Primary author**	**Abad-Corpa, E**[44]	**Caley, L**[38]	**Cronin, M**[45]	**Daley, B**[39]	**Fagan, D**[62]	**Fairweather, C**[61]	**Felton, B**[76]	**Fonville, A**[53]
**Theory used**	Participatory action research design from a qualitative methodological perspective, using Checkland’s ‘Soft Systems’ theoretical framework	Participatory action research design	Rootman et al., Freire *et al.*	Linking new to past experiences, probing deeply in past experiences	The research study was undertaken via a constructivist paradigm.	The study was guided by the methodology of constructivism. This approach to qualitative inquiry is based on the assumption that in order to gain an understanding of the social world we need to examine it from the perspective of those who arc the active participants in that world.	The case study described in this paper used a ‘constructivist’ methodology, that is, a research technique that utilizes key actors’ and close observers’ understandings and interpretations of the implementation (Guba and Lincoln, 1989).	Constructivist paradigm
**Aspects of theory used**	Social environment plays a critical role in the development of knowledge.	Social environment plays a critical role in the development of knowledge.	Social environment plays a critical role in the development of knowledge.	Learning is a result of the individual’s interaction with the environment	Cognitive dissonance as the stimulus for learning	Learning is a result of the individual’s interaction with the environment	Learning is a result of the individual’s interaction with the environment	Learning is a result of the individual’s interaction with the environment
**KTA phase**	Step 4: Select, tailor and implement intervention	Step 4: Select, tailor and implement intervention	Step 4: Select, tailor and implement intervention	Step 3: Assessing barriers and facilitators	Step 3: Assessing barriers and facilitators	Step 3: Assessing barriers and facilitators	Step 2: Adapting knowledge to local context	Step 1: Identify problem
**Primary author**	**Lipman, T**[59]	**Smith, C**[42]	**Tilleczek, K**[43]	**Varpio, L**[82]	**Greenslade, 2010**[63]	**McWilliam, 2009**[79]	**Rogers, 2011**[64]	**Schluter, 2011**[60]
**Theory used**	Constructivism approach	Learning Constructivism Theory	Contructivism approach - general	Constructivist grounded theory	Constructivist approach with in-depth interviews and comparative analysis to develop and systemically organize data into four major interrelated themes and a connecting essential thread.	Constructivism approach	A constructivist grounded theory approach was adopted for this study on the basis that it would allow for the use of sensitising concepts or guiding interests derived from the conflict literature, as well as an investigation of the features of conflict unique to the OR team.	Situated within a constructivist methodology that considered individual experiences, abilities, and knowledge in the construction of scope of practice
**Aspects of theory used**	Social environment plays a critical role in the development of knowledge.	Learning is a result of the individual’s interaction with the environment	Learning is a result of the individual’s interaction with the environment	Learning is a result of the individual’s interaction with the environment	Learning is a result of the individual’s interaction with the environment	Learning is a result of the individual’s interaction with the environment	Social environment plays a critical role in the development of knowledge.	Learning is a result of the individual’s interaction with the environment
**KTA phase**	Step 1: Identify problem	Step 4: Select, tailor and implement intervention	Step 4: Select, tailor and implement intervention	Step 3: Assessing barriers and facilitators	Step 3: Assessing barriers and facilitators	Step 1: Identify problem	Step 2: Adapting knowledge to local context	Step 3: Assessing barriers and facilitators
**Primary author**	**Adler, R**[46]	**Carr, S**[54]	**Field, D**[55]	**Greenhalgh,T**[65]	**Higgs, J**[56]	**Holtslander, L**[77]	**Hoshmand, L**[51]	**Hunter, J**[40]
**Theory used**	Theories and definitions of evidence based on Descartes, Locke's theory of ‘tabula rasa; Hume, von Uexkull - Merk-Mal theory; Ginzburg; Glaserfeld's understanding of constructivism in knowledge; clinical examples to illustrate models of organisms (Richter; Wolf and Wolff)	Highlights the potential value and contribution of hermeneutic phenomenology and constructivist approaches to exploring and knowing nursing as a means to addressing some of the practice learning challenges	learning is a mental process, in terms of the con-structivist view of learning or whether it owes more to enculturation into social processes as with the situated learning and legitimate peripheral participation approaches to learning	Although we believe the constructivist approach has general validity, it is particularly appropriate for the promotion of the knowledge and skills for knowledge translation.	In this paper, the critical question of knowledge as the underpinning of clinical practice is examined. The nature of knowledge is explored in this paper, with support being given to the constructivisit perspective	Constructivism	Constructivism (Berger and Luckmann, 1966; Bruffee, 1986; K. J. Gergen, 1985) calls for multiple paradigms of knowledge. The potential of multiple rationalities and methods of construction is recognized by the cognitive interpretation of science	Constructivist learning theory was an appropriate conceptual framework for the course as it acknowledges multiple, socially constructed truths, perspectives, and realities versus a single reality
**Aspects of theory used**	All 3 aspects	Learning is a result of the individual’s interaction with the environment	Social environment plays a critical role in the development of knowledge.	Learning is a result of the individual’s interaction with the environment	All 3 aspect	All 3 aspects	All 3 aspects	All 3 aspects
**KTA phase**	Knowledge creation: knowledge synthesis	Knowledge creation: knowledge inquiry	Knowledge creation: knowledge synthesis	Step 4: Select, tailor and implement intervention	Knowledge creation: knowledge inquiry	Knowledge creation: knowledge synthesis	Step 1: Identify problem	Step 4: Select, tailor and implement intervention
**Primary author**	**Kinsella, E**[57]	**Labonte, R**[48]	**Lyddon, W**[78]	**McGuckin, C**[58]	**Miller, K**[50]	**Neimeyer, R**[80]	**Plack, M**[49]	**Rogal, S**[41]
**Theory used**	The constructivist perspective is founded on the idea that humans actively construct their personal realities and create their own representational models of the world’	This article argues further that a ‘constructivist’ research paradigm not only has the potential to resolve some of the tensions between research and practice in health promotion but also is inclusive of knowledge generated by the conventional paradigm.	Constructivism approach in general	Constructivist learning is based on an eclectic mix of ideas derived primarily from cognitive neuroscience including information processing theory.	Constructivism emphasizes the socially constructed nature of reality; it shifts attention away from the search for universal truths and toward an exploration of what is considered real within particular social contexts.	In sharp contrast to this worldview, social constructivism endorses a form of postmodernism (Anderson, 1990) that turns nearly every aspect of this modern psychological program on its head. Gone is the faith in an objectively knowable universe, and with it the hope that elimination of human bias, adherence to canons of methodology, and reliance on a pure language of observation would yield a ‘true’ human science, mirroring psychological reality without distortion.	The constructivist emphasizes the personal meaning made by the inquirer and the inquired.	Constructivism relates to the philosophy that the meaning of new learning is constructed upon current knowledge
**Aspects of theory used**	Cognitive dissonance as the stimulus for learning	Cognitive dissonance as the stimulus for learning	Cognitive dissonance as the stimulus for learning	Social environment plays a critical role in the development of knowledge	Learning is a result of the individual’s interaction with the environment	All 3	All 3	Learning is a result of the individual’s interaction with the environment
**KTA phase**	Knowledge creation: knowledge synthesis	Knowledge creation: knowledge synthesis	Knowledge creation: knowledge tools/products	Step 3: Assessing barriers and facilitators	Knowledge creation: knowledge inquiry	Knowledge creation: knowledge inquiry	Knowledge creation: knowledge synthesis	Step 4: Select, tailor and implement intervention
**Primary author**	**Rolloff, M**[81]	**Wilson, H**[51]	**Appleton, J.**[47]					
**Theory used**	Constructivism assumes that learners construct knowledge as part of a process of making sense of their experiences: ‘Learners, therefore, are not empty vessels waiting to be filled, but rather active organisms seeking meaning’ (Driscoll, 2005, p. 387).	The underlying science here is located in a constructivist philosophy while other descriptive terms would be phenomenological, interpretivist or subjectivist	Philosophical underpinnings of constructivism, post-positivism, critical realism (in terms of realistic evaluation) and participatory inquiry					
**Aspects of theory used**	Learning is a result of the individual’s interaction with the environment	Social environment plays a critical role in the development of knowledge.	All 3					
**KTA phase**	Knowledge creation: knowledge synthesis	Step 3: Assessing barriers and facilitators	Knowledge creation: knowledge inquiry					

### Stages of the knowledge-to-action cycle

As shown in Table 5, 13 studies involved knowledge creation (n = 7 knowledge synthesis, n = 5 knowledge inquiry and n = 1 knowledge tools). Twenty-two studies addressed one of the four specific steps of the action cycle: four studies addressed step one, ‘identify the problem or knowledge gap’, two studies addressed ‘adapting knowledge to local context’ (step two), and the remaining were equally divided between step three ‘assessing barriers and facilitators’ (n = 8) and step four ‘select, tailor and implement intervention’ (n = 8). No study mapped onto more than one step of the action cycle.

### Thematic analysis

We identified five themes related to the applications of social constructivist theory in KT with several nested concepts within each theme (Table 6).

**Table 6:** Main themes and major concepts emerging from the application of social constructivist theory to knowledge translation interventions

**THEME 1**	**THEME 2**	**THEME 3**	**THEME 4**	**THEME 5**
**Meaning of ‘evidence’**	**Understanding acquisition, expression and application of knowledge in and for professional practice**	**Promoting professional expertise as a component of evidence-based practice**	**Understanding clients and their experiences**	**Designing interventions to a) increase knowledge and skill acquisition; b) change behaviour**
Post-modernist views on knowledge and knowledge acquisition	Practice based on experience	Knowledge of ethics and professional practice	Outcomes regarding patient care	For generating EBP knowledge
Original/authentic problems to be addressed	Role of personal and professional values vs. formal knowledge	Novice vs. expert	Understanding of clients’ realities	For sharing knowledge
Meaning of evidence	Experiential learning	Progress and role		For impacting on knowledge, attitudes and intentions to apply o evidence in practice
	Meaning of experiences	SC in how learning and expertise develop		For promoting reflective practice
	Patient welfare as a motivator	Differences between specialist and generalist in skills and knowledge		For problem solving, critical thinking and reflection
	Perceived support	Combining experiences		For changing attitudes
	Loyalty to profession	Working together		For creating meaning
	Learner satisfaction and involvement			For sharing knowledge (Theoretical and practical)
	Meaning of competency			For knowledge that is actionable
	Role of previous experience			For practice based evaluations
	Direct, reflective learning			
	Role of context			
	Feedback from colleagues			
	Learners guide learning process			
	Threat of evaluation			

### Theme one: meaning of evidence and tension between research and practice (n = 9 papers)

The papers [46-52,76,79] in this theme reported findings from literature reviews exploring the meaning of evidence and the epistemology of research and practice. Papers in this theme recognized that there may be various definitions of ‘knowledge’ and ‘knowledge creation’ which may vary depending upon the theoretical lens used to explore the applications of knowledge in clinical practice. Adler’s [46] review of the history of science literature suggested that different types of evidence can and should be used in health research. Appleton [47] discussed the relevance of constructivism to researchers in health services while Labonte’s [48] literature review on the social constructivist paradigm in health promotion research, suggested that this paradigm has the potential to resolve some of the philosophical tensions between research and practice in health promotion. Plack [49] suggested that physical therapists should shift their focus from mainly positivist approaches to care to more constructivist ones in order that they may make better use of evidence. In another literature review, Miller [50] found that the social constructivist paradigm could serve as a bridge between researchers and practitioners by suggesting that research efforts be directed towards identifying the needs of those who will be offering and receiving health care services. Wilson [51] discussed the biomedical model of care and introduced a debate on the effectiveness of objectivism in health care. The authors suggested that a more subjectivist model to healthcare, and one that embraces social constructivist theories, would include recent evidence on doctor-patient relationship as a major contributor to patient outcomes in addition to incorporating ‘objective’ clinical findings. Hoshmand’s [52] literature review emphasized a broader choice of research methods, the development of reflective skills in practice and better linkages between researchers and practitioners.

### Theme two: understanding of acquisition, expression and application of knowledge in and for professional practice (n = 14 papers)

Social constructivism was used as a lens through which to gain a greater understanding of how knowledge is acquired, manifested and used to inform practice as well as to explain the individual and contextual factors that have an impact on skill development and/or behavior [39,53-60,77,78,80-82]. Nine of the 14 papers corresponding to this theme were literature reviews, conceptual papers and reflective pieces [54-58,77,78,80,81]. Topics were varied and ranged from how factors such as loyalty to the profession has an impact on practice [53], to how tailoring information to practice needs influences learning and learner satisfaction [39].

Major findings from the review papers included the notion that the social constructivist paradigm provides a means for professionals to voice their knowledge [54], the importance of context in learning and the difficulty in transferring knowledge to different contexts in physical therapy practice [55]. Higgs [56] explored the nature of knowledge and discussed knowledge as ‘underpinning clinical practice’. He suggested that knowledge is an active and dynamic phenomenon constantly undergoing changes and being tested in practice. In addition, Higgs’ paper emphasized that knowledge is the basis for evidence-based practice, as clinicians draw from experiential and declarative sources of knowledge in their daily practice. Kinsella [57] discussed the philosophical underpinnings of reflective practice and how these can be used to advance our knowledge and interpretation of practice. McGuckin’s [58] literature review focused on the most effective methods for teaching modern psychiatric practice knowledge, attitudes, and skills. The author found that constructivist learning is an eclectic approach with great potential as learners are actively engaged in the learning process and they bring their unique perspectives to the learning situation. Moreover, relevant and meaningful learning activities are used to promote the desired knowledge and skills. A qualitative study of physician researchers found that implementing research evidence is more complex than suggested in current models of evidence-based medicine and that clinical decision-making is strongly influenced by factors other than just research evidence [59]. Schluter [60] used a critical incident technique grounded in a constructivist methodology to understand how nurses conceive their scope of practice. Findings suggested that different nursing areas of expertise have diverse scopes of practice, that require varied methods for applying knowledge, with the optimal method relying on nurses’ grade and skill mix.

### Theme three: promoting professional expertise as a component of evidence-based practice (n = 1 paper)

Fairweather [61] used focus groups with primary healthcare nurse specialists in order to identify the characteristics and attributes of competency that specialist nurses ascribe to their practice; describe how specialist nurses delineate specialist boundaries from generalist practice; and generate evidence based knowledge for the development of regulatory procedures for nurses. Results indicated that knowledge is a synthesis of propositional and practice knowledge and that expertise was gained through exposure and reflection. Assumptions from social constructivist theory were used to help novices move towards expertise in practice. Differences in knowledge acquisition and application were believed to be associated with level of experience and expertise.

### Theme four: understanding clients and their experiences/realities (n = 2 papers)

This theme focused on the use of constructivist approaches for examining the ‘centrality’ of the patient. The two studies in this theme found that health care professionals keep the patient at the ‘center’ of their clinical decisions and treatment interventions when acquiring and applying knowledge. Fagan [62] used a constructivist inquiry approach to examine accident and emergency nurses’ perceptions of their roles in identifying child abuse in primary healthcare. The ability of nurses to identify children in potentially abusive situations required nurses to evaluate the child’s safety as accurately as possible, to ensure that he/she received the appropriate treatment and attention. The author concluded that although all nurses in the study had sufficient knowledge to identify child abuse, this knowledge base increased with experience. The author also suggested that additional training and education is needed for multidisciplinary decision making about the role of nurses in this context. A qualitative study by Greenslade and Mandville-Ansey [63] used in-depth interviews within a constructivist approach to understand the experiences of women having same-day breast cancer surgery. Women’s subjective experiences were used to make client-centered recommendations to assist healthcare professionals in effecting change to enhance quality of care.

### Theme five: designing interventions aimed at knowledge and skill acquisition and changing behavior (n = 9 papers)

Nine studies examined the effects of various interventions aimed at increasing knowledge and skills in order to improve practice. One study used a mixed methods design [44], one used a qualitative design [64] and the remaining seven used surveys [38,42,43,45] and workshop evaluations [40,41,65]. The intervention studies assessed workshops that were related to specific clinical training skills such as cultural competence [40] and alcohol dependence screening [38], while some focused on social constructivism approaches as strategies for experiential learning and reflective practice [45]. In this theme, social constructivist theory was used to inform the design of KT interventions intended to promote core skills, knowledge, and competencies needed for evidence based practice, and support behavior change (increased use of best practices).

## Discussion

The purpose of this scoping review was to examine the applications of social constructivist theory in knowledge translation for best practice in the health professions. Consistent with the findings by Colquhoun *et al.*[19] and Davies *et al.*[20], the use of social constructivist theory in the KT literature is limited and haphazard. Most papers describing results of original research neglected to justify why the use of theory was central to the research question, and most papers did not make explicit the relevance and potential applications of social constructivist theory in KT. While we acknowledge that lack of justification for theory use represents a major limitation of the papers, most (n = 28) were published after Colquhoun *et al.*’s [19] and Davies *et al.*’s [20] review papers. Likewise, 23 papers were published before the germinal articles by Eccles *et al.*[14] and the ICEBerg group [15]. Indeed these KT scholars have advocated for explicit statements regarding the use of theory in KT research [14,15,17,19,20,66-68]. We suggest that without at least a definition of the theory and at most, a discussion of theoretical assumptions and underpinnings, the potential for theories to guide and inform the field of KT is limited at best.

There was important variability in the study designs, areas of practice, targeted health professions, and methodological approaches used across the 35 papers. The papers ranged from reflective discussions to qualitative studies, and only six papers used an experimental design to assess the impact of various KT interventions grounded in social constructivist approaches. Such variability most likely reflects the early stages of development in the use of social constructivism as a potentially valuable theory in the field of KT.

In terms of practice settings, we were interested in the clinical areas where the theory would be most frequently applied. Twenty-six papers reported results from five different types of clinical settings and practice areas (ten papers were from the primary healthcare setting, seven from postgraduate education environments, six from mental health service settings, one from home care and two from health services organizations). It is not clear whether this represents a heterogeneous list of settings and as such, we cannot conclude on the nature of the clinical settings most often targeted by efforts to highlight the link between social constructivism and KT.

Among the health disciplines represented in the reviewed papers, nursing accounted for one-third. This finding is consistent with the current state of KT research in this profession. Indeed, nursing scholars have produced much of the seminal KT literature, including key papers in KT theory [20,69]. While eight of the 35 papers addressed KT within interdisciplinary teams, few studies mentioned which other professions were involved or their roles in the KT interventions. Overall, there was representation from seven disciplines reflecting the broad spectrum of professions interested in KT and best practice.

Although we were able to identify which components of the KTA framework [32] were targets of the study interventions and/or discussions (Table 6), these were not addressed explicitly in any of the papers. An assumption of the KTA framework believed to be important for both researchers and practitioners is that it considers various sources of information as ‘knowledge’ and/or ‘evidence’. These sources include knowledge from research findings as well as other forms of knowing such as experiential knowledge [32], which refers to learning from experience through reflection and is considered essential for integrating and making sense of the knowledge that emerges from scientific research [70].

According to Graham and Tetroe [34], the KTA falls within the social constructivist paradigm as it ‘privileges social interaction and adaptation of research evidence that takes local context and culture into account…and offers a holistic view of the KT phenomenon by integrating the concepts of knowledge creation and action’. The framework underscores the fluid boundaries between knowledge creation and application as it highlights the need to create knowledge that emerges from knowledge users’ questions (KTA step one), when it emphasizes the need to adapt the knowledge to the local context (KTA step two), and when it suggests that we select, tailor, and implement interventions that will facilitate the uptake of new knowledge for practitioners (KTA step four). Indeed, the authors of the KTA framework advocate for a participatory model whereby end users (*e.g.*, clinicians, multidisciplinary teams, patients, and decision makers) are involved in developing research questions and carrying out research activities [32]. Collaborative interactions at every step of the KT process are believed to facilitate optimal use of research evidence and other forms of knowledge in clinical practice [71-73]. The KTA framework has the potential to help researchers understand the mechanisms that promote a collaborative approach to identifying knowledge gaps and the likelihood of successful interventions aimed at changing practice.

The final discussion points relate to the main contributions of social constructivism in the field of KT in the health professions. The focus on the meaning of evidence across health professions continues to be a dominant issue in the literature. In fact, this review has highlighted that post-modernist views of knowledge, knowledge acquisition, and knowledge construction do support the legitimacy of the various sources of evidence and their use in practice. Constructed knowledge or knowledge that emerges from a collaborative constructive process among various stakeholders in the clinical setting, can and should be considered as valid sources of evidence in conjunction with research generated evidence.

A major finding from the review involves the acquisition, expression, and application of knowledge in practice, with an emphasis on how social constructivist perspectives support clinicians in expressing this knowledge in their professional interactions. The important role of ‘context’ in promoting and supporting best practices, which has been emphasized by several KT scholars [32,70,74,75], was also highlighted in this review. There is a clear need for future targeted research, as researchers and practitioners grapple with identifying and addressing the complex interaction of individual and contextual variables that play a key role in clinical practice. In their 1995 paper, Higgs and Titchen suggested that knowledge is active and dynamic, constantly undergoing changes and being tested in practice [56]. The notion that knowledge is dynamic in nature is congruent with the major tenets of social constructivism and should be reflected in the evaluation process of knowledge translation interventions [24,27,28]. We suggest that this idea must be assigned its rightful place in the KT discourse [31] as the dynamic nature of knowledge creation and exchange is also consistent with the core principles of the KTA framework.

Least surprising and perhaps most important, are the findings from the final theme: the use of social constructivist assumptions for designing and implementing interventions aimed at knowledge and skill acquisition and behavior change. As is the case with many other learning theories, designing environments and interventions that will foster optimal learning and behavior change represents educational best practice [16,17,66]. Indeed, relying on constructivist assumptions to support the design of KT interventions designed to foster changes in knowledge, skills, attitudes, and behavior is a practice that has been strongly advocated [14,15,17,66].

### Limitations

Scoping reviews offer a unique opportunity to retrieve and scan a broad range of literature to answer a research question. A potential limitation is that by searching the titles, abstracts, and subject headings only, we may have missed relevant papers. We could have also used citation chaining in Web of Science or Scopus to find other articles that cited the key articles we had already found.

We recommend that future research in this area clearly cite the theories used to design KT interventions to ensure that they are identified in reviews similar to this one, as well as in systematic reviews of theory.

The articles included in this review were not appraised for their scientific rigor, as scoping reviews do not typically include critical appraisals of the evidence. In deciding to summarize and report the overall findings without the scrutiny of a formal appraisal process, we recognize that our results speak to the extent of the research activity, major conclusions and research gaps, rather than provide the reader with support for the effectiveness of interventions or for evidence-informed recommendations that were grounded in the social constructivist paradigm. However, this represents the evolution of research that explicitly incorporates social constructivist theory in the development and application of KT. As a larger number of studies rigorously test KT interventions guided by theory, systematic review and quality appraisal will be necessary.

## Conclusion

This review is the first to examine the use of social constructivism in KT studies. Results from this review contribute to discussions that are currently taking place on the use and usefulness of theory in KT. There are divided opinions about the value of theory in the field and about whether it is possible to have overarching theories that can be used to further solidify the science of KT, improve practice, and ultimately improve patient care. Despite the debates over this issue, we argue that moving forward without considering the use of theory in KT is not sound scientific practice. Without theory, it will be difficult to understand the underlying mechanisms behind interventions, understand the impact the various interventions have on behaviour change and to compare across studies.

Our review indicates that social constructivism has not been widely explored in the field of KT. As a sociological theory of knowledge, it has the potential to illuminate how individuals use new information and knowledge to make sense of existing practices and how the meaning of the new knowledge may change as a result of an individual’s existing knowledge base and the relevance of the new knowledge to existing practices.

We argued that the links between social constructivism and KT have not been fully explored but that the KTA framework has constructivist underpinnings that help the discussion on how the theory can be used in the broader KT enterprise moving forward. Indeed the KTA framework advocates for interventions that consider learning to be the result of human interactions that take place within a socially mediated context.

There will undoubtedly be many more discussions and debates about using theories to advance the science of KT and perhaps even more conversations about which theories are most appropriate in a given situation or context. This will become increasingly important as our knowledge base about the likelihood of successful interventions across contexts and health disciplines continues to grow. We suggest that social constructivist theories hold much promise for informing the design of KT interventions. In this context, social constructivist interventions will take into account that practicing clinicians are part of complex social systems, that they may privilege active collaborative strategies for knowledge acquisition and that the potential for learning is greater when the new knowledge conflicts in some way with existing practices (cognitive dissonance). Moreover, KT interventions grounded in social constructivist theories will value clinicians’ prior knowledge and experiences as essential components of knowledge creation and application. These critical sources of ‘evidence’ will be in constant interaction with new knowledge and scientific evidence to help support clinicians in developing new understandings of clinical phenomena.

Finally, we propose that further research is needed to test the use of social constructivist assumptions in the design and implementation of KT interventions. The contribution of the theory lies in its potential to unveil the individual processes that are involved in the ‘construction’ and application of knowledge in clinical practice.

## Competing interests

The authors declare that they have no competing interests.

## Authors contributions

All authors made substantial contributions to conception and design, or acquisition of data, or analysis and interpretation of data. All authors were also involved in drafting and revising the manuscript for important intellectual content. All authors have given final approval of the version to be published.

## Funding

This scoping review was supported by the Edith Strauss Rehabilitation Research Projects, a knowledge translation initiative funded by the Richard and Edith Strauss Foundation of Canada and supported by the School of Physical and Occupational Therapy, McGill University. The following present committee members contributed to the project (some of which are authors of this manuscript): Sara Ahmed PhD, Genevieve Côté-Leblanc OT, Erika Hasler OT, Nicol Korner-Bitensky PhD, Annette Majnemer PhD, Nancy Mayo PhD, Ana Maria Rodriguez PhD, Anita Menon PhD, Laurie Snider PhD, Aliki Thomas PhD, and Diana Valentini PT. We would like to acknowledge Lucy Li for her assistance in formatting this manuscript and Drs. Ian Graham and Andre Bussieres for their valuable feedback on earlier versions of the manuscript.

## Supplementary Material

Additional file 1 Full electronic search strategy for Ovid Medline (1948 – May 16, 2011). Click here for file
