# Supplementary material for: Applications of social constructivist learning theories in knowledge translation for healthcare professionals: a scoping review
Source: Implement Sci. 2014 May 6;9:54. doi: 10.1186/1748-5908-9-54 (PMC4040365; doi:10.1186/1748-5908-9-54)
Supplement: Additional file 1 — Full electronic search strategy for Ovid Medline (1948 – May 16, 2011). [file 1748-5908-9-54-S1.docx]

**Additional file 1:**

**Full electronic search strategy for Ovid Medline (1948 – May 16, 2011)**

1 constructivi*.mp.

2 exp Clinical Competence/ or exp Knowledge/ or exp Information Dissemination/ or knowledge translation.mp. or exp"Diffusion of Innovation"/ or exp Health Knowledge, Attitudes, Practice/

3 exp Evidence-Based Medicine/ or evidence-based practice.mp.

4 Quality improvement.mp. or Quality Improvement/

5 dissemination.tw.

6 organi*ational innovation.mp.

7 (implementation adj3 research).mp.

8 research utili*ation.mp.

9 complex intervention.mp.

10 Practice Guidelines as Topic/ or exp Guideline Adherence/ or guideline* adherence.mp.

11 Professional Practice/ or exp Physician's Practice Patterns/ or practice pattern*.mp.

12 exp Education, Medical/ or exp Education, Continuing/ or exp Competency-Based Education/ or exp Education, Professional/ or exp Education, Distance/ or exp Education, Professional, Retraining/ or exp Education, Medical, Continuing/

13 competenc*.mp. or exp Competency-Based Education/

14 Physical Therapy Modalities/ or "Physical Therapy (Specialty)"/

15 (physical therap* or physiotherap*).mp.

16 occupational therapy/ or occupational therap*.mp.

17 Nursing Education Research/ or exp Education, Nursing/ or Evidence-Based Nursing/

18 exp Physicians/

19 exp Nurses/

20 (health care professional* or health professional*).mp.

21 Learning/ OR "Attitude of Health Personnel"/ OR Models, Educational/ OR nursing/ OR nursing methodology research/ OR nursing models/ OR "Outcome Assessment (Health Care)"/ OR Health Services Research/

22 or/2-21

23 1 and 22

#New subject headings found from analysis of 30 selected citations from original group
